# Critical transitions in rainfall manipulation experiments on grasslands

**DOI:** 10.1002/ece3.6072

**Published:** 2020-02-06

**Authors:** Ilaíne S. Matos, Bernardo M. Flores, Marina Hirota, Bruno H. P. Rosado

**Affiliations:** ^1^ Ecology and Evolution Graduate Program IBRAG Rio de Janeiro State University Rio de Janeiro Brazil; ^2^ Department of Plant Biology University of Campinas Campinas Brazil; ^3^ Department of Physics Federal University of Santa Catarina Florianopolis Brazil; ^4^ Department of Ecology IBRAG Rio de Janeiro State Univiersity Rio de Janeiro Brazil

**Keywords:** alternative stable states, critical transitions, experimental drought, net primary productivity, rain‐out shelter, regime shifts

## Abstract

As a result of climate and land‐use changes, grasslands have been subjected to intensifying drought regimes. Extreme droughts could interfere in the positive feedbacks between grasses and soil water content, pushing grasslands across critical thresholds of productivity and leading them to collapse. If this happens, systems may show hysteresis and costly management interventions might be necessary to restore predrought productivity. Thus, neglecting critical transitions may lead to mismanagement of grasslands and to irreversible loss of ecosystem services. Rainfall manipulation experiments constitute a powerful approach to investigate the risk of such critical transitions. However, experiments performed to date have rarely applied extreme droughts and have used resilience indices that disregard the existence of hysteresis. Here, we suggest how to incorporate critical transitions when designing rainfall manipulation experiments on grasslands and when measuring their resilience to drought. The ideas presented here have the potential to trigger a perspective shift among experimental researchers, into a new state where the existence of critical transitions will be discussed, experimentally tested, and largely considered when assessing and managing vegetation resilience to global changes.

## Graphical abstract

We suggest two different approaches of how rainfall manipulation experiments in grasslands can be carried out to investigate critical transitions of productivity. We aim to trigger a perspective shift and guide researchers along the trajectory to a new state, where the existence of critical transitions will be discussed, experimentally tested, and largely considered when assessing and managing vegetation resilience to global changes.

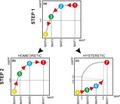

## 1 EXTREME DROUGHTS MAY TRIGGER CRITICAL TRANSITIONS IN GRASSLAND PRODUCTIVITY

**Figure 1:**
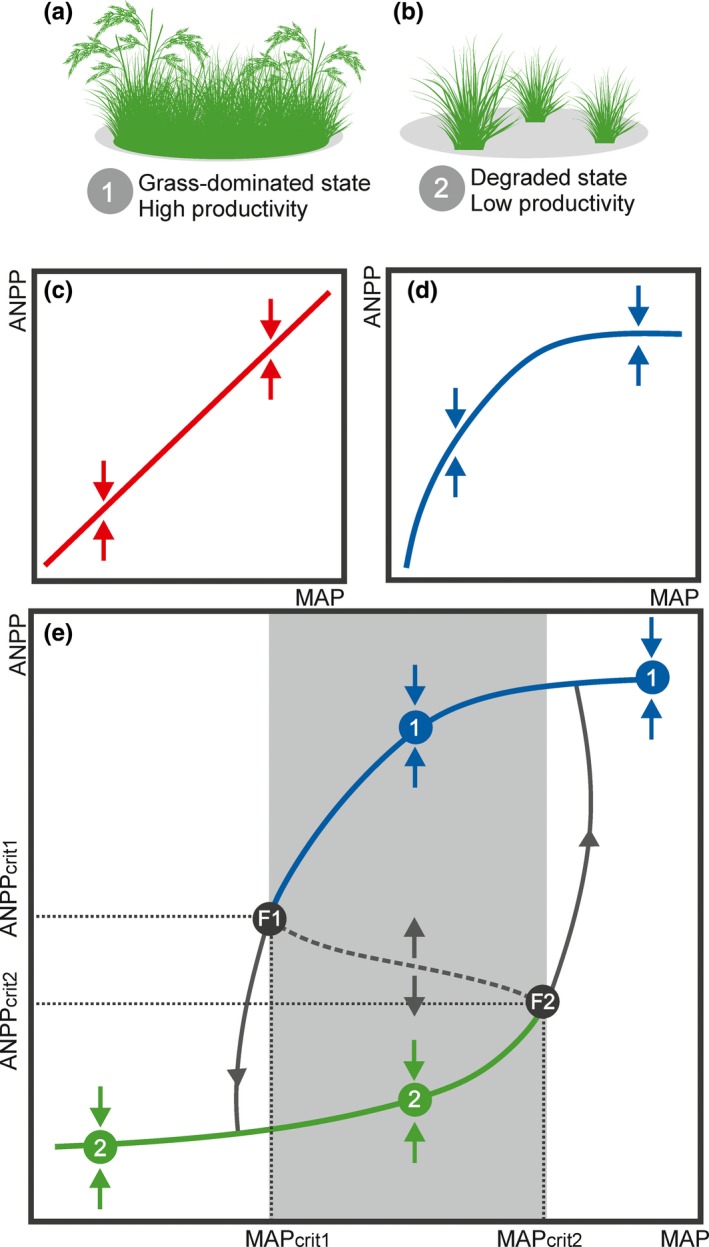
Searching for hysteresis in grassland ecosystems. Alternative stable states in grassland productivity (a,b) and conceptual models (c–e) for the relationship between above‐ground net primary productivity (ANPP) and mean annual precipitation (MAP). (a) High‐productivity state: At high MAP, grasses form a nearly continuous layer and sustain higher productivity (high ANPP); (b) low‐productivity state: At lower MAP, grass layer becomes sparser and sustain lower productivity (low ANPP); (c) linear model: The system changes gradually; (d) nonlinear model: The system changes abruptly; (e) discontinuous model: The system presents two alternative stable states (bistability); 1. high‐productivity state; 2. low‐productivity state; MAP_crit1_—critical precipitation threshold for collapse (below this threshold grasslands shift from state 1 to 2); MAP_crit2_—critical precipitation threshold for grass re‐establishment (above this threshold grasslands shift from state 2 to 1); ANPP_crit1_—critical productivity threshold for collapse (below this threshold grasslands shift from state 1 to 2); ANPP_crit2_—critical productivity threshold for grass re‐establishment (above this threshold grasslands shift from state 2 to 1); and gray area—range of MAP values where the two alternative stable states can coexist. Arrows indicate the direction of change if the system is not in equilibrium, that is, after being displaced from its initial position by a disturbance, the system tends to converge to the stable state within the current basin of attraction (solid lines) and diverge from the unstable state (dashed line)

**Figure 2:**
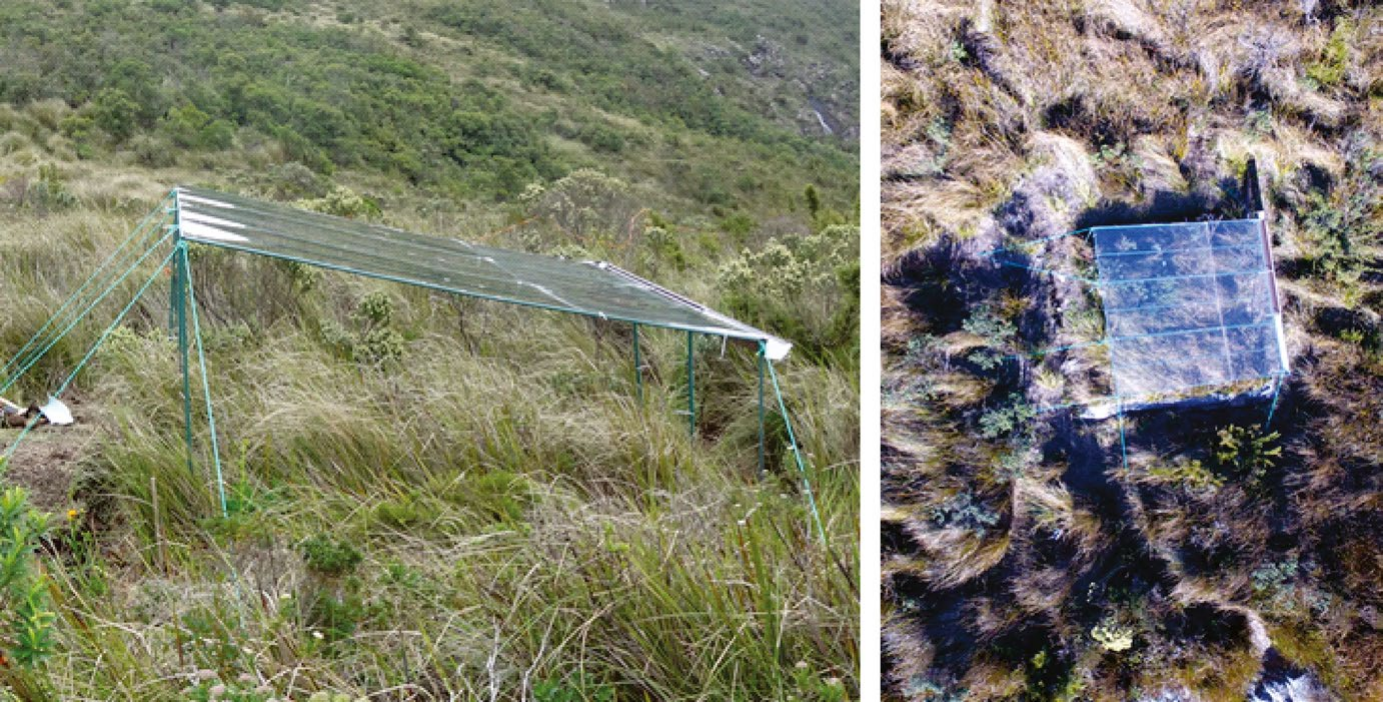
Example of rain‐out shelter used to conduct rainfall manipulation experiments, installed in a tropical mountain grassland in Brazil (photograph credit: Ilaíne S. Matos and Fabio Takashi)

**Figure 3:**
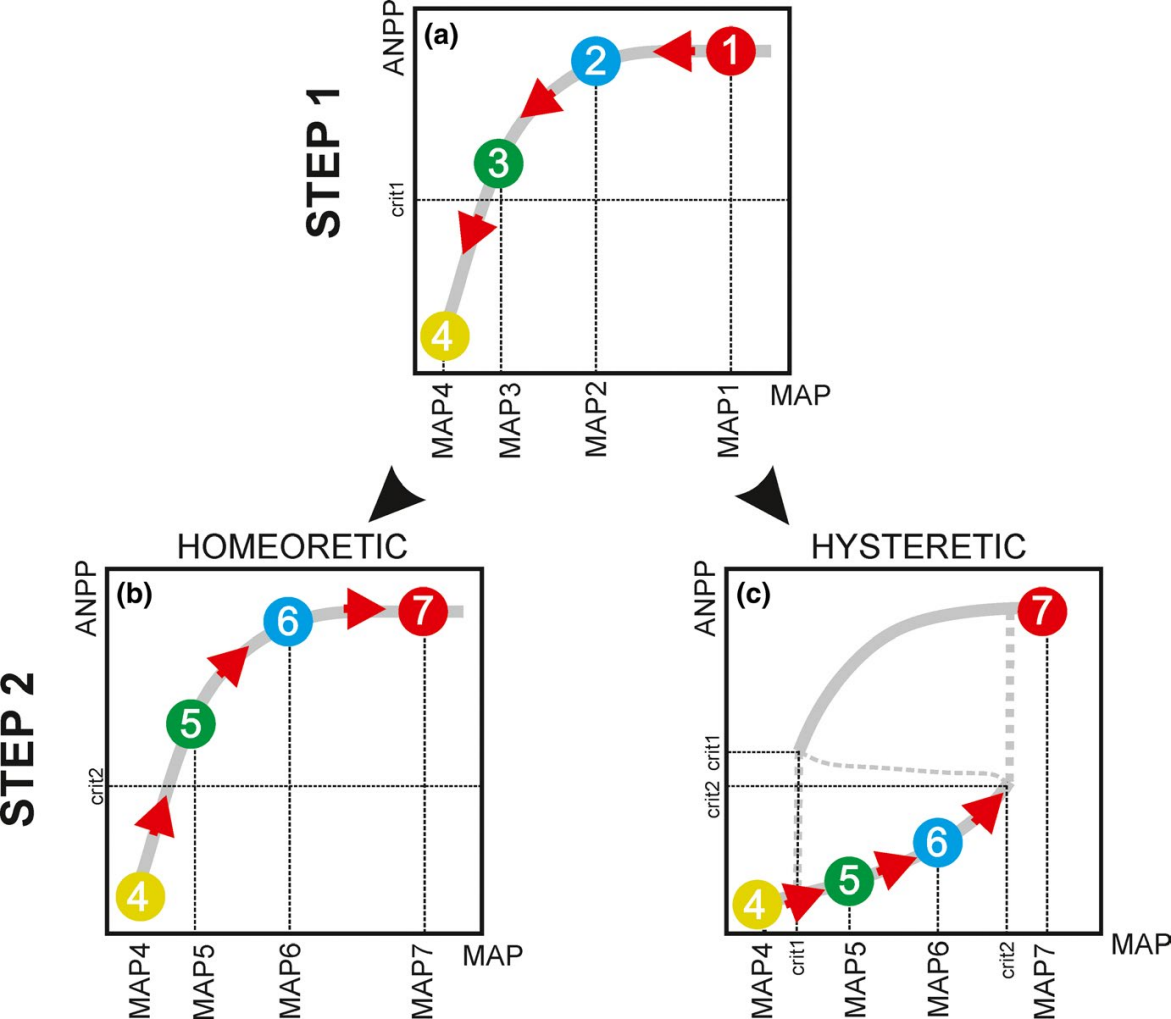
Testing for hysteresis in rainfall manipulation experiments. (a) STEP 1—Subjecting grasslands to progressive declines in mean annual precipitation (MAP): Rain‐out shelters are installed to fully intercept rainfall (intercept 100% of precipitation) and drought treatments are irrigated to receive progressively less rainfall at each growing season (MAP1—MAP4), until the system crosses the critical threshold of productivity (ANPP_crit1_) and collapses; (b,c) STEP 2—Subjecting grasslands to progressive increases in MAP (i.e., drought reversal experiment): grasslands are irrigated in order to gradually increase MAP conditions at each growing season (MAP4—MAP7), until the system bounces back to the high‐productivity state (i.e., crosses ANPP_crit2_); (b) in homeoretic systems (without critical transitions), the forward and backwards trajectories are roughly the same; (c) in hysteretic systems (with critical transitions), those trajectories are different because the critical precipitation threshold for grass re‐establishment (MAP_crit2_) is higher than the critical threshold for its collapse (MAP_crit1_)

As droughts become longer, more frequent and intense (Donat, Lowry, Alexander, O'Gorman, & Maher, 2016), emerges the need to understand how ecosystems may respond to varying precipitation regimes. This is especially important for grasslands as they have a global distribution, covering ca. 18% of the Earth terrestrial surface (Dixon, Faber‐Langendoen, Josse, Morrison, & Loucks, 2014) and contributing up to 30% of terrestrial gross primary productivity (Forrestel et al., 2017). Grassland above‐ground net primary productivity (ANPP) is mainly determined by precipitation (Knapp & Smith, 2001) and under wetter conditions grasses usually form a nearly continuous layer, resulting in high productivity (Figure 1a). When the mean annual precipitation (MAP) declines, grasslands may shift persistently to a less productive state (Figure 1b). If only observational records of ANPP versus MAP are considered, such transitions appear approximately linear (Figure 1c, and also see figure 2 in Sala, Gherardi, Reichmann, Jobbagy, & Peters, 2012). However, when experimental data from both irrigation and drought experiments are included, a nonlinear relationship emerges, with productivity saturating under wetter conditions and reducing abruptly under moderate drought (Figure 1d, and also see figure 3 in Knapp, Ciais, & Smith, 2017).

Those linear and nonlinear models of ANPP versus MAP have been useful to predict and optimize biomass production over short periods and under near‐normal conditions (Knapp et al., 2017). However, they may not capture grassland responses to extreme drier conditions (Gao, Zhang, Tang, & Wu, 2019). Extreme droughts (see Glossary in Table 1) may push the system across critical thresholds of productivity (ANPP_crit1_), triggering discontinuous transition from a high‐productivity state to an alternative low‐productivity state (Figure 1e). Such critical transitions may happen, for instance, when stressing conditions reduce diversity and redundancy within the system, thus decreasing its resilience to droughts and others disturbances (Folke et al., 2004). While in the linear and nonlinear models transitions are gradual, continuous, and homeoretic (i.e., changes in the system state follow similar path, Figure 1c,d), this may not be case for grassland‐rainfall systems. Grassland dynamics might present bistability (e.g., Isbell, Tilman, Polasky, Binder, & Hawthorne, 2013; Miller, Belote, Bowker, & Garman, 2011; Porensky, Mueller, Augustine, & Derner, 2016), that is, two alternative stable states (with either high or low/null productivity), separated by critical thresholds (MAP_crit_ and ANPP_crit_), and hence, they can show hysteresis (i.e., deviations away and back toward a starting point are different, Figure 1e).

**Table 1:** Glossary

Term	Definition
Alternative stable state	Two or more equilibrium states to where a system can converge after undergoing a disturbance or perturbation
Bistable systems	Systems where for the same value of the condition variable (e.g., MAP) two alternative stable states can coexist (e.g., high‐ and low‐productivity states)
Condition variable	Any biotic, physical, or resource factor that drives the state of a system (e.g., MAP and grass cover)
Critical transition	Transitions between alternative stable states
Critical threshold	Unstable state or condition of the system in which any small deviation may push it to an alternative stable state
Critical productivity threshold for degradation	Value of ANPP (ANPP_crit1_) below which the system shifts from the high‐ to the low‐productivity state
Critical productivity threshold for plant re‐establishment	Value of ANPP (ANPP_crit2_) above which the system shifts from the low‐ to the high‐productivity state
Critical precipitation threshold for degradation	The value of MAP below which the system shifts from the high‐ to the low‐productivity state (MAP_crit1_)
Critical precipitation threshold for plant re‐establishment	The value of MAP above which the system shifts from the low‐ to the high‐productivity state (MAP_crit2_)
Disturbance	Discrete event in time that changes the condition variable and then displaces the system away from its equilibrium state (e.g., drought)
Extreme droughts	Drought events whose intensity, frequency, and/or duration fall outside the range experienced by the organisms composing a given community along their evolutionary history
Homeoretic system	System where deviations away and back toward a starting point are similar
Hysteretic system	System where deviations away and back toward a starting point are different, because forward and backward trajectories are described by distinct functions
Monostable systems	Systems where for each value of the condition variable (e.g., MAP) there is only one stable state
Rain‐out shelter	Structure used in rainfall manipulation experiments to intercept different percentages of precipitation, thus simulating distinct levels of drought treatments (intensity, frequency, duration, and/or seasonality)
Recovery	System capacity or tendency to return to its initial state after undergoing a disturbance
Recovery time	Time required for the system to return to its initial state
Resilience	The system ability to remain in the same state, with similar functioning, despite disturbances that push it closer to critical thresholds
Response diversity	The diversity of responses exhibited by the components of a certain community when subjected to disturbances
State variable	Variable that describes and constitutes the system (e.g., ANPP)

Critical transitions may cause biomass to decline abruptly, constraining grassland ability to provide important ecosystem services, such as carbon storage and forage provision for livestock (Forrestel et al., 2017). Moreover, restoring grasslands to the initial and desired high‐productivity state may require costly efforts, as hysteresis implies that a return to the original precipitation regime would not necessarily push the system back to its predrought ANPP state (Scheffer, Carpenter, Foley, Folke, & Walker, 2001), that is, in systems with hysteretic behavior the critical precipitation thresholds for collapse (MAP_crit1_ in Figure 1e) and for grass re‐establishment (MAP_crit2_ in Figure 1e) might differ.

The main reason for such hysteretic responses is the presence of positive feedbacks between grasses and their environment (Scheffer et al., 2001). Arguably, the most important one is the feedback by which grasses improve soil–water availability for their own growth (D'Odorico, Okin, & Bestelmeyer, 2012). Therefore, in the high‐productivity state the presence of a nearly continuous grass layer reduces soil evaporation and water run‐off, thus increasing soil water content and self‐reinforcing the current state (more grass = more soil–water = more grass). As conditions become drier, grass coverage decreases, causing evaporation and run‐off to increase. At MAP_crit1_ the grass layer is too sparse to ameliorate soil–water availability and conditions become too harsh for plant recolonization. Consequently, grasslands collapse and become trapped in a low‐productivity state (less grass = less soil–water = less grass), implying that much wetter conditions (MAP_crit2_) are required for the system to bounce back to its original productivity (i.e., to cross ANPP_crit2_; Holmgren et al., 2006).

Identifying the critical thresholds (ANPP_crit_ and MAP_crit_) is then of utmost importance to guide management policies and to maintain grassland productivity and other important ecosystem services under drier conditions (Carpenter, Brock, Folke, Nes, & Scheffer, 2015; Miller et al., 2011). Rainfall manipulation experiments are a powerful approach to test the existence of such critical thresholds, because they allow the assessment of vegetation response to precipitation reduction far below the current ranges (i.e., extreme droughts). Hundreds of rainfall manipulation experiments have been conducted across the world (Hoover, Wilcox, & Young, 2018), particularly in grasslands (Matos, Menor, Rifai, & Rosado, 2019), because it is relatively easier to install rain‐out shelters in such open environments (Figure 2), compared to shrublands and forests. Furthermore, grassland responses are usually quicker and easier to monitor (e.g., Hoover et al., 2018; Ruppert et al., 2015).

Even though some of those experiments have reported significant structural changes in species composition and abundances, they have not been designed to specifically test for alternative stable states of productivity (Kreyling, Jentsch, & Beier, 2013; Luo, Jiang, Niu, & Zhou, 2017; Smith, Knapp, & Collins, 2009). Actually, most of them have failed to subject grasslands to extreme drought treatments and to monitor biomass production after drought cessation (Estiarte et al., 2016; Gao et al., 2019; Matos et al., 2019). Additionally, most studies have assessed resilience using metrics (Ingrisch & Bahn, 2018) that disregard the possibility that alternative stable states may exist in the system (Kéfi et al., 2019; van Nes et al., 2016). Hence, we have little mechanistic information about what shapes grassland resilience and whether they can undergo critical transitions in response to extreme droughts.

## 2 INCORPORATING CRITICAL TRANSITIONS INTO RAINFALL MANIPULATION EXPERIMENTS

**Figure 4:**
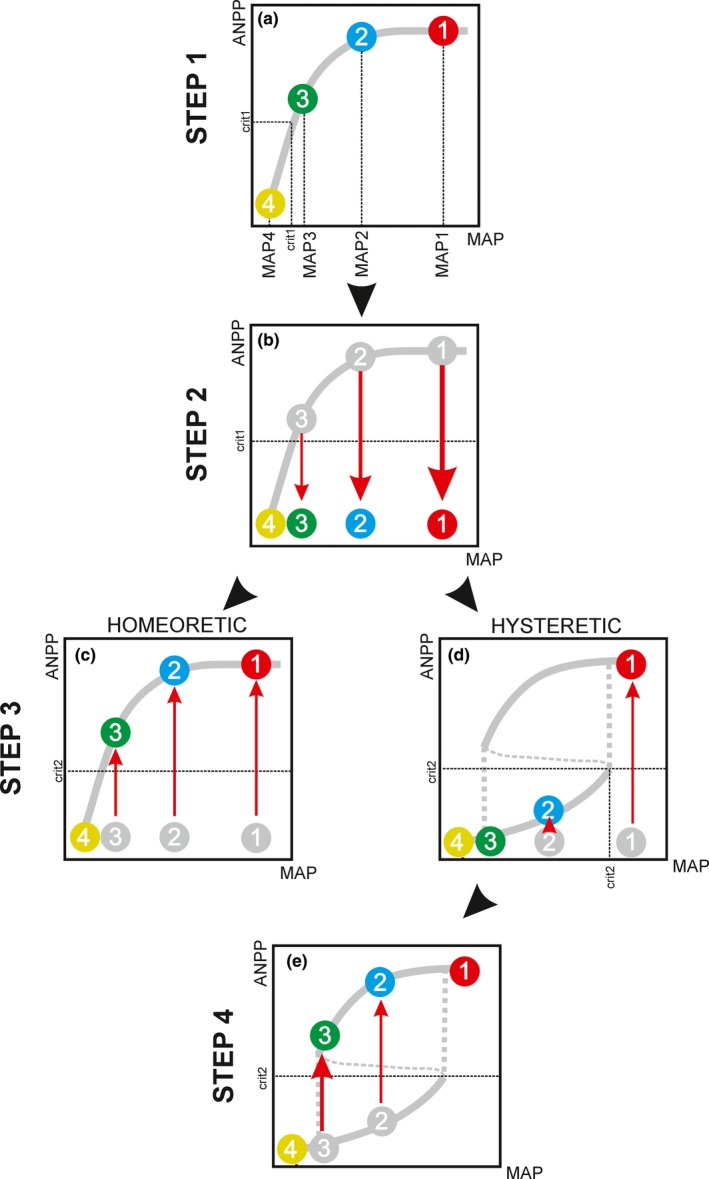
Testing for nonrecovery in rainfall manipulation experiments. (a) STEP 1—Subjecting grasslands to a wide range of reduced levels of initial mean annual precipitation (MAP): Rain‐out shelters are installed to fully intercept precipitation and then drought plots are irrigated to receive distinct percentages of reduced MAP (MAP1—MAP4). Grassland at level 4 is subjected to a MAP condition below the critical precipitation threshold (MAP1 < MAP_crit1_) and undergoes a state shift from high to low productivity state (i.e., it crosses the critical above‐ground net productivity threshold—ANPP_crit1_); (b) STEP 2—Disturbing grasslands until they shift to a new state: For the plots that do not undergo a state shift in the first step (1–3), irrigation can be completely stopped until all plots collapse. Thinner red arrows indicate that lower levels of disturbance (e.g., less consecutive days without rain) are required to trigger a state shift as the system is closer to the critical threshold; (c–d) STEP 3—Removing disturbances and testing for nonrecovery: After the collapse, plots are irrigated again to receive the distinct levels of MAP (as in Step 1) and postdisturbance productivity is monitored in all plots; in systems without critical transitions (c), all plots are expected to recover their predisturbance above‐ground net primary productivity state (ANPP), while in systems with critical transitions (d), recovery only occurred at level 1 (outside the bistability range), which was subjected to MAP conditions above the critical precipitation threshold for plant re‐establishment (MAP1 > MAP_crit2_); (e) STEP 4—Disturbing grasslands until they shift back to the initial state: Levels 2 and 3 are irrigated to push the system back to its initial state (i.e., cross the ANPP_crit2_). Thicker red arrows indicate that higher levels of disturbance (e.g., longer periods of irrigation) were required to trigger a state shift

Inspired by previous studies in laboratorial (e.g., Dai, Vorselen, Korolev, & Gore, 2012; Veraart et al., 2012) and field conditions (Isbell et al., 2013; Schmitz, 2004), we suggest two different experimental approaches to investigate critical transitions of productivity in grasslands: testing for hysteresis (Figure 3) and testing for nonrecovery (Figure 4).

To test for hysteresis, grasslands must be firstly subjected to progressive declines in MAP at each growing season (i.e., 10%, 25%, 50%, 80% reduction in MAP), thus leading to gradual declines in ANPP (Figure 3a). Eventually, the system would cross a critical threshold of productivity (ANPP_crit1_) and collapse into the low‐productivity state (level 4 in Figure 3a). After the collapse, a drought reversal experiment (Ratajczak et al., 2017) could be conducted, by progressively increasing MAP through irrigation (Figure 3b,c). Such gradual increments in MAP should lead to small increases in ANPP, until the system reaches a second critical threshold (ANPP_crit2_) and bounces back to the high‐productivity state (level 7 in Figure 3b,c). Using this experimental approach, one may describe ANPP responses to both increases and decreases in MAP, hence differentiating between systems with homeoretic (without critical transitions) and hysteretic (with critical transitions) behaviors (Scheffer & Carpenter, 2003; Schroder, Persson, & Roos, 2005). In homeoretic systems, forward and backward trajectories would occur roughly at the same MAP (Figure 3b). In hysteretic systems, however, those trajectories would differ (Figure 3c), as the critical precipitation threshold for grass re‐establishment (MAP_crit2_) would be higher than the critical threshold for collapse (MAP_crit1_).

When conducting tests for hysteresis, researchers must be aware of the effects of interannual variations in precipitation on drought treatments (Matos et al., 2019). For instance, if the experiment is conducted in a grassland with MAP = 1,200 mm and during the first experimental year—that is when precipitation should be decreased by 10%—a natural drought event reduces total precipitation to 1,000 mm, then the actual precipitation received in the drought treatments would be 900 mm, instead of 1,080 mm. To overcome this problem, precipitation should be fully intercepted (i.e., rain‐out shelters intercepting 100% of precipitation) in both control and drought plots. Then, control plots should be irrigated to maintain constant precipitation levels that correspond to the historical precipitation recorded in the study area (e.g., MAP = 1,200 mm), while drought plots should receive the desired reduced precipitation amount along the experiment (e.g., 1,080 mm in the first year = 10% reduced MAP).

Another crucial point is that the rate of change of the condition variable (MAP) must be lower than that of the state variable (ANPP). Otherwise, some hysteresis‐like pattern could be observed even in homeoretic systems (Scheffer & Carpenter, 2003). So, in grasslands composed by annual species, changes in MAP could be conducted incrementally at each growing season. However, in grasslands dominated by perennial species, it could take 10–20 years (Heisler & Weltzin, 2006; Valone, Meyer, Brown, & Chew, 2002) for the system to stabilize in the new MAP condition before the next level of MAP reduction could be applied. Consequently, long‐term (multidecadal) experiments would be required to identify hysteresis in perennial grasslands.

An alternative approach to evaluate critical transitions in those systems would be the test for nonrecovery (i.e., collapse, Figure 4). In this case, instead of gradual changes in MAP across years, grasslands are simultaneously subjected to a wide range of reduced MAP values (MAP1—MAP4, Figure 4a). So, in the plots with lowest reduction of MAP (MAP4), ANPP may fall below the ANPP_crit1_, causing the system to collapse. Thus, this experimental approach would allow identifying the range of MAP values within which the system has alternative stable states and may undergo a state transition from the high‐ to the low‐productivity state.

For the plots that did not collapse in the previous step, that is, persisted in the original high‐productivity state even after equilibration with the reduced MAP condition (1–3 in Figure 4a), stronger disturbances, such as extreme drought events, could be applied to try to force the system across the ANPP_crit1_ (Step 2 in Figure 4). Extreme drier conditions could be applied, for example, by completely stopping irrigation (i.e., 100% reduction in MAP) until the system collapses (Figure 4b). Besides extreme droughts, other types of disturbances, such as fire and grazing, could also be experimentally tested, in isolation or in combined factorial treatments (Smith, 2011). It would be especially interesting to combine different levels of precipitation and grazing, as this approach could provide valuable information for grassland management (von Wehrden, Hanspach, Kaczensky, Fischer, & Wesche, 2012). Those experiments would allow the investigation of, for each value of MAP reduction, how much grazing pressure grasslands could endure before suffering a shift to an undesirable and low‐productivity state.

After all, plots have collapsed into the low‐productivity state (Figure 4b), disturbances are ceased (i.e., MAP conditions restored to initial values), and the recovery of productivity must be monitored (Figure 4c,d). Productivity may recover to its predisturbance values in all plots (Figure 4c), or may recover in some plots (i.e., plot 1 with MAP > MAP_crit2_, Figure 4d), but not in others (i.e., plots 2 and 3 with MAP < MAP_crit2_, Figure 4d), thus suggesting the existence of critical transitions (bistability) in the latter case.

For a proper identification of bistability, the experiment described above must satisfy two important conditions. First, system recovery must not be prevented by isolation from sources of propagules (Isbell et al., 2013; Scheffer & Carpenter, 2003), that is, after disturbances have ceased, adequate conditions for regeneration must be provided in all plots, such as having nearby intact grasslands, or by applying active grass planting. Otherwise, even homeoretic systems would not be able to recover their productivity (Connell & Sousa, 1983). Second, recovery must be monitored for sufficient time to allow the system to stabilize to the new condition (Schroder et al., 2005). For instance, if the ANPP is recovering at a slow rate, and recovery is not monitored for sufficient time, one may interpret that the system has stabilized in the low‐productivity state, when it is actually on a slow backward journey (Porensky et al., 2016). It has been suggested that recovery should be monitored for at least one complete community turnover, that is, for enough time to ensure that all the initial resident individuals of the system have replaced themselves (Connell & Sousa, 1983). The time for a complete turnover can be approximated to the life span of the longest living species present in the community (Schroder et al., 2005), which can vary from one year (annual grasses) up to two decades (perennial grasses; Heisler & Weltzin, 2006; Valone et al., 2002). Sometimes, however, the distinction between lack of recovery and slow recovery is impractical or unnecessary. For instance, if the system is frequently disturbed it may not achieve full recovery, persisting trapped in the low‐productivity state. In such cases, for management purposes, interventions to push a system back to the desirable high‐productivity state would be required, either for a grassland that is recovering too slowly or for one that is not recovering at all (Isbell et al., 2013).

Experiments designed to test for nonrecovery may actually provide a great opportunity for investigating which management interventions could be applied to restore grassland productivity after it has collapsed. Although wetter conditions are usually required for the system to bounce back, too much rain, especially if concentrated in a few events, may worsen soil erosion instead of enabling plant recolonization (Holmgren et al., 2006; Reichstein et al., 2013). Therefore, in a final experimental step (Figure 4e), multiple levels of irrigation could be applied across the plots to determine which range of precipitation values would promote grass re‐establishment and which would reinforce soil degradation. As the occurrence of extreme droughts followed by heavy rainfall events may become more frequent in the future (Donat et al., 2016), those experiments would provide valuable information for grassland management policies.

## 3 ASSESSING RESILIENCE IN BISTABLE GRASSLANDS

**Figure 5:**
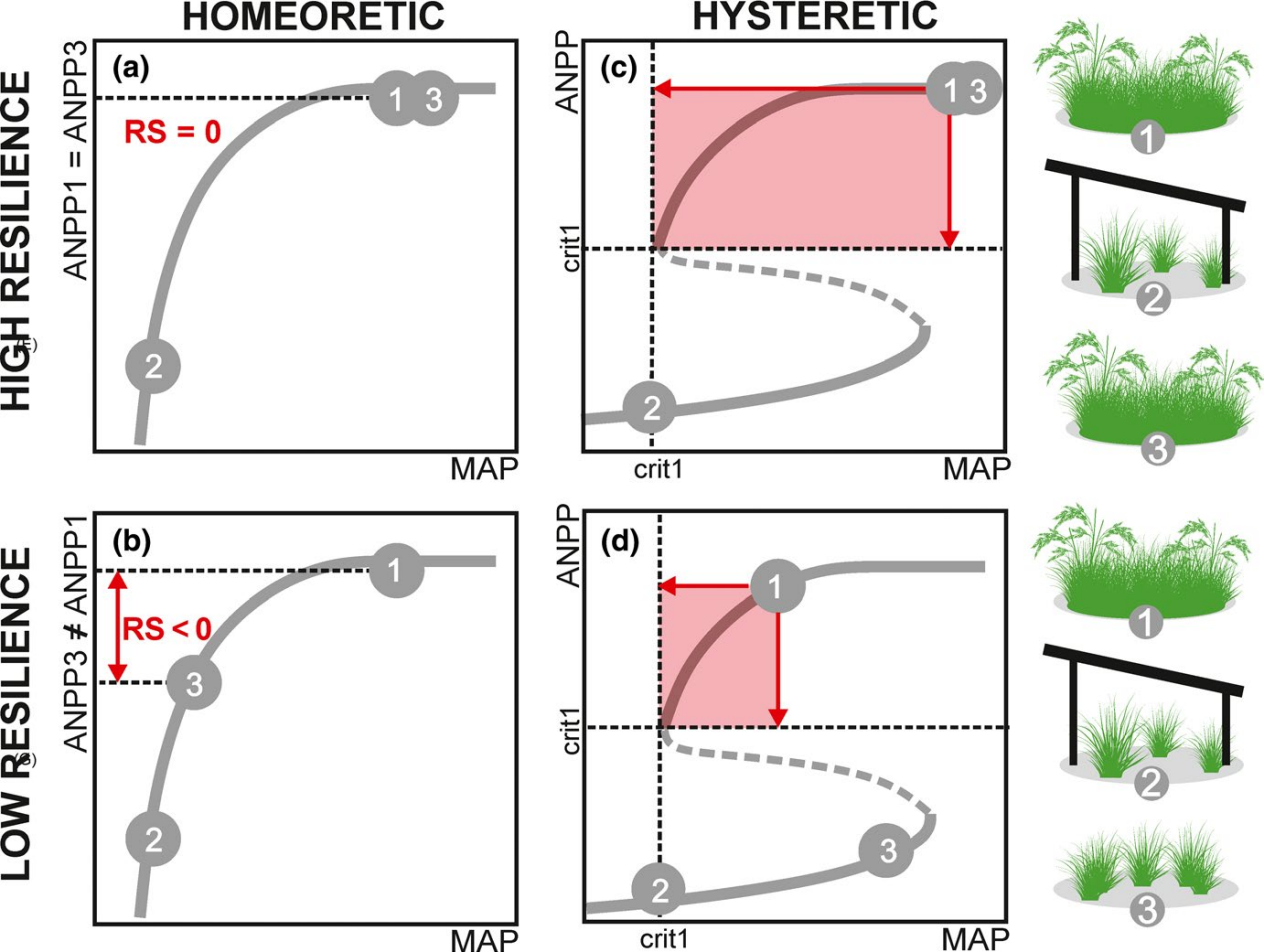
Measuring resilience of grassland productivity in response to experimental droughts. Grasslands are subjected to an experimental drought and above‐ground net primary productivity is measured before drought (ANPP1), immediately after drought cessation (ANPP2) and after the recovering period (ANPP3); (a–b) in systems without critical transitions (homeoretic), resilience (RS) can be assessed by comparing ANPP1 and ANPP3. In (a), recovery was monitored for sufficient time, so that ANPP values before and after drought did not significantly differ (ANPP1 = ANNP3) and the system is considered resilient (RS = 0). In (b), recovery was not monitored for sufficient time, so productivity after drought was lower than before drought (ANPP3 < ANPP1) and the system is considered nonresilient (RS < 0); (c–d) in systems with critical transitions (hysteretic and bistable), resilience can be assessed as the system distance from critical thresholds of precipitation (e.g., MAP_crit1_) and productivity (e.g., ANPP_crit1_). The area delimited by the distanced to those thresholds is larger for systems with high resilience (c) than for systems with low resilience (d)

In rainfall manipulation experiments conducted to date, resilience has often been assessed by measuring how close the system is from its initial state (Ingrisch & Bahn, 2018; Kéfi et al., 2019; Smith, Wilcox, Power, Tissue, & Knapp, 2017). This is usually done by comparing ANPP values obtained before drought (ANPP1), immediately after drought cessation (ANPP2) and after the recovery period (ANPP3; Figure 5a,b; Matos et al., 2019). The closer ANPP3 values are from ANPP1, the more resilient the system is (i.e., in Figure 5a ANPP1 = ANPP3, thus RS = 0). In homeoretic systems, the ANPP will always tend to return to predrought levels, as long as disturbances are ceased and conditions are restored too (Scheffer & Carpenter, 2003). Consequently, according to this approach, if recovery is monitored for sufficient time, even grasslands that seem initially nonresilient (RS < 0, Figure 5b) may eventually reach their predrought ANPP and achieve resilience (RS = 0).

In bistable systems (Figure 4c,d), however, grasslands may remain trapped in the low‐productivity state, unless they are subjected to MAP values above the critical precipitation threshold for grass re‐establishment (MAP > MAP_crit2_; Scheffer & Carpenter, 2003). In this case, instead of measuring how close the system is from its initial state, resilience may be assessed by measuring how close the system is from the critical thresholds (Holling, 1973). So, resilience can be measured as the system distance to MAP_crit _or ANPP_crit_ thresholds. The closer the system is to such thresholds, the less resilient it is, as it is more likely to collapse if disturbed. Recently, the area delimited by the distances to both thresholds has been proposed as an integrative metric to compare resilience across systems (Ciemer et al., 2019), with larger areas indicating higher resilience (Figure 5c,d).

In experiments designed to test for nonrecovery, resilience could also be estimated as the magnitude of disturbance beyond which recovery is no longer possible (Holling, 1973). In Figure 4, for example, the magnitude of disturbance could be assessed as the number of consecutive days without rain, and thus, resilience would increase from level 4 to 1 (i.e., more consecutive days without rain are necessary to trigger a critical transition in level 1 than in level 4).

Finally, the recovery time is another indicator of resilience (Dai, Korolev, & Gore, 2015). As a system approaches the critical threshold, longer time is required for a return to the initial state (i.e., recovery time increases). If the threshold is crossed, recovery time tends to infinite (i.e., the system does not return to its initial state; Dai et al., 2015; Veraart et al., 2012). Therefore, in homeoretic systems (Figure 4c) longer recovery times would be expected for systems subjected to lower initial MAP reductions (recovery time 1 > 2 > 3 > 4), whereas in bistable systems (Figure 4d) recovery times for plots subjected to initial MAP below MAP_crit2_ would tend to infinite.

## 4 CONCLUDING REMARKS

Here, we suggest two innovative approaches for designing rainfall manipulation experiments in grasslands, which may allow assessing the risk of critical transitions in ecosystem productivity. In the first approach (test for hysteresis), state transitions are triggered by gradual changes in MAP, which may reveal hysteretic patterns of ANPP responses. This experimental approach, however, could depend on long‐term experiments for perennial grasslands, which have a much slower dynamics than annual grasslands. For perennial systems, we propose a second approach (test for nonrecovery), where disturbances are applied to force faster state transitions. In this case, although forward and backwards trajectories (hysteresis) may not be identified in detail, it is still possible to investigate the risk of critical transitions by monitoring if productivity recovers to predrought values, or instead, if the system remains trapped in the low‐productivity state. In addition, we explain how resilience can be measured in both homeoretic and hysteretic systems.

By conducting such experiments in several types of grasslands (e.g., tropical, desert, and temperate), it would be possible to decipher ecosystem‐specific responses and the mechanisms driving changes in ANPP. For example, one could estimate how critical thresholds of precipitation and productivity differ between drier and wetter grasslands (e.g., Are desert grasslands more likely to undergo critical transitions than mesic grasslands?). Similarly, by monitoring other state variables besides ANPP, such as plant eco‐physiological tolerance, plant functional traits, and community composition, one could determine their relative importance for ecosystem stability (e.g., How changes in species diversity and abundance contribute to ANPP stability in response to drought?). In this case, such experiments could eventually lead us to discover that grasslands can exhibit not only two, but, perhaps, multiple alternative stable states (Seabloom & Richards, 2003).

Finally, a deeper understanding of how grasslands behave in response to drought would allow societies to manage them more accurately (Briske, Fuhlendorf, & Smeins, 2003; Miller et al., 2011; Suding & Hobbs, 2009). If extreme droughts can indeed trigger critical transitions in grasslands, they would persist in the unproductive state indefinitely, unless the drought event is followed by unusual wetter years and/or managers intervene in the system (Holmgren et al., 2006). In this case, management practices should focus on avoiding collapses by enhancing the resilience of the high‐productivity state. Resilience could be promoted, for example, by sowing a variety of plant species, or by applying small disturbances (e.g., periodically inducing droughts and/or controlled burns). Both practices would potentially result in the coexistence of species with distinct strategies to cope with drought (i.e., higher response diversity) and, then, in a higher resilience status not only to droughts, but also to other types of disturbances (Carpenter et al., 2015). If those practices fail and a collapse occurs, management should be re‐oriented to erode the resilience of the undesirable state, for example, by improving conditions for plant re‐establishment through irrigation, sowing, seedling plantation, and soil‐improvement (Holmgren et al., 2006).

Although we focused on grasslands and droughts, we highlight that the two experimental approaches proposed can also be applied to other ecosystems and can be forced by other external conditions besides rainfall (e.g., raising temperatures and CO_2_ concentrations). Therefore, we hope the ideas present here can trigger a perspective shift and guide researchers along the trajectory to a new state, where the existence of critical transitions will be discussed, experimentally tested, and largely considered when assessing and managing vegetation resilience to global changes.

## CONFLICT OF INTERESTS

We have no competing financial, professional, or personal interests to declare.

## AUTHORS' CONTRIBUTIONS

B.H.P. Rosado, B. M. Flores, I.S. Matos, and M. Hirota conceived the idea. I.S. Matos led the writing of the manuscript. All authors contributed critically to the drafts and gave final approval for publication.

## Data Availability

Data sharing not applicable to this article as no datasets were generated or analyzed during the current study.
